# Reactive Oxygen Species: Modulators of Phenotypic Switch of Vascular Smooth Muscle Cells

**DOI:** 10.3390/ijms21228764

**Published:** 2020-11-20

**Authors:** Adnan Badran, Suzanne A. Nasser, Joelle Mesmar, Ahmed F. El-Yazbi, Alessandra Bitto, Manal M. Fardoun, Elias Baydoun, Ali H. Eid

**Affiliations:** 1 Department of Nutrition, University of Petra, P.O. Box 961343, Amman 11196, Jordan; abadran@uop.edu.jo; 2 Department of Pharmacology and Therapeutics, Beirut Arab University, P.O. Box 11-5020, Beirut 1107-2809, Lebanon; san413@bau.edu.lb; 3 Department of Biology, American University of Beirut, P.O. Box 11-0236, Beirut 1107-2020, Lebanon; jm104@aub.edu.lb; 4 Department of Pharmacology and Toxicology, American University of Beirut, P.O. Box 11-0236, Beirut 1107-2020, Lebanon; ae88@aub.edu.lb; 5 Department of Pharmacology and Toxicology, Alexandria University, Alexandria 21526, Egypt; 6 Department of Clinical and Experimental Medicine, University of Messina, 98125 Messina, Italy; abitto@unime.it; 7 Department of Basic Medical Sciences, College of Medicine, QU Health, Qatar University, Doha P.O. Box 2713, Qatar; 8 Biomedical and Pharmaceutical Research Unit, QU Health, Qatar University, Doha P.O. Box 2713, Qatar

**Keywords:** cardiovascular disease, phenotypic switch, reactive oxygen species, vascular smooth muscle cell

## Abstract

Reactive oxygen species (ROS) are natural byproducts of oxygen metabolism in the cell. At physiological levels, they play a vital role in cell signaling. However, high ROS levels cause oxidative stress, which is implicated in cardiovascular diseases (CVD) such as atherosclerosis, hypertension, and restenosis after angioplasty. Despite the great amount of research conducted to identify the role of ROS in CVD, the image is still far from being complete. A common event in CVD pathophysiology is the switch of vascular smooth muscle cells (VSMCs) from a contractile to a synthetic phenotype. Interestingly, oxidative stress is a major contributor to this phenotypic switch. In this review, we focus on the effect of ROS on the hallmarks of VSMC phenotypic switch, particularly proliferation and migration. In addition, we speculate on the underlying molecular mechanisms of these cellular events. Along these lines, the impact of ROS on the expression of contractile markers of VSMCs is discussed in depth. We conclude by commenting on the efficiency of antioxidants as CVD therapies.

## 1. Introduction

Reactive oxygen species are oxygen byproducts of metabolic reactions taking place in the cell. They include superoxide anion (O_2_^−^), hydrogen peroxide (H_2_O_2_) and hydroxyl radical (HO^•^). Superoxide anions are produced in a controlled manner by NADPH oxidases. They undergo dismutation by superoxide dismutase (SOD), leading to the production of hydrogen peroxide. In turn, hydrogen peroxide may be converted to HO^•^, a highly reactive ROS. At the cellular level, ROS play the role of secondary messengers of signaling pathways that underlie key events, such as cell differentiation, growth and death [1]. In addition, ROS are implicated in several physiological processes, such as the regulation of vasotone, immune responses, and others [2,3]. Notably, an imbalance between pro- and anti-oxidants leads to exaggerated ROS production [4]. This increase in ROS levels results in oxidative stress, which induces damage to cellular components such as DNA, lipids and proteins [5,6]. Furthermore, the resulting disturbance in the cellular redox balance mediates the pathogenesis of many diseases [4,7]. 

An increasing body of evidence shows that oxidative stress is strongly involved in the pathophysiology of cardiovascular diseases (CVD), including hypertension, atherosclerosis, aortic aneurysms and vascular restenosis [8,9,10]. In fact, NAD(P)H oxidases (NOX), which are expressed in vascular cells [9], are responsive to many chemical stimuli, such as angiotensin II (Ag-II), physical stimuli, including mechanical stretch and pressure, and hypoxia [7,11]. Consequently, the activation of these enzymes leads to excessive ROS production [12]. The resulting oxidative stress sets the stage for CVD by reducing the bioavailability of nitric oxide (NO), promoting endothelial dysfunction and altering vascular response [9]. Importantly, oxidative stress induces vascular smooth muscle cell (VSMC) proliferation and migration, thus contributing to atheroma formation and restenosis. 

VSMCs are crucial components of blood vessels and the major determinants of vasotone [13,14]. This critical and tightly regulated function is granted by the contractile phenotype of VSMCs [13,14,15]. In response to certain cues, VSMCs switch to a synthetic dedifferentiated phenotype characterized by increased proliferative and migratory capabilities [16,17]. In addition, synthetic VSMCs show an increased secretion of extracellular matrix (ECM) proteins [16,17].

Several factors that modulate VSMC phenotypic switch have been reported. These include growth factors such as transforming growth factor (TGF)-β [18] and fibroblast growth factor (FGF) [19], cytokines such as monocyte chemokine protein 1 (MCP-1) [20], and endothelial peptides such as endothelin-1 [21]. Prostaglandin D_2_ [22], microRNAs [23], hyperhomocysteinemia [24] and cyclic stretch [25] have emerged as more recent non-canonical modulators. Estrogen, especially by virtue of its ability to increase the intracellular pool of cAMP, has also been shown to modulate VSMC function, and thus phenotype [26,27,28,29,30]. Indeed, in addition to its role in promoting vasorelaxation [31], cAMP has been shown to modulate the expression of adrenergic receptors as well as cellular phenotypes [26,32,33,34,35,36]. Importantly, this cAMP, which can act through its downstream PKA or Epac pathways [37,38], elicits the aforementioned effects in microvascular smooth muscle cells mainly via Epac [26,32,37,38]. 

A rather controversial modulator of VSMC phenotypic switch is ROS [39]. In fact, the literature presents some inconsistency regarding the role of ROS in VSMC differentiation. While a substantial number of studies report a proliferative effect of ROS on VSMCs, thus inducing a dedifferentiated phenotype [40], other studies show that ROS significantly increases the expression of VSMC differentiation markers [41]. 

Several sources of ROS in VSMCs have been reported [42,43,44]. These include mitochondrial respiratory chain, xanthine oxidase, lipoxygenases and NOXs [42,43,44]. Indeed, NOX1 and NOX4 are expressed in the VSMCs of large arteries, while NOX2 is expressed in the VSMCs of resistance and coronary arteries [45,46]. In addition, NOX5 has been found to be expressed in the VSMCs of human aorta [47]. Interestingly, vascular NOXs differ in their subcellular localizations, responsiveness to agonists, and amount of ROS released [42].

In this review, we highlight the effect of ROS on VSMC phenotype, a critical determinant of vascular function and physiology [29]. Specifically, we examine the major hallmarks of VSMC phenotypic switch, namely cell proliferation, migration and expression of contractile markers. In addition, we speculate on the underlying signaling pathways and factors accounting for the differential response of VSMCs to ROS. 

## 2. Effect of ROS on VSMC Proliferation and Migration

Cell proliferation is a major hallmark of VSMC phenotypic switch [16]. The effect of ROS on this cellular process has been overwhelmingly documented [7]. Contextually, most reports indicate that ROS promote VSMC proliferation [7,8,48,49]. Furthermore, ROS mediate the proliferative effects of hormones and growth factors on VSMCs. For instance, H_2_O_2_ facilitates the proliferative effects of bradykinin, Ag-II and growth factors such as platelet-derived growth factor (PDGF) and thrombin [50,51,52,53,54], whereas O_2_^−^ mediates plasminogen urokinase-induced VSMC proliferation [55].

**Figure 1:**
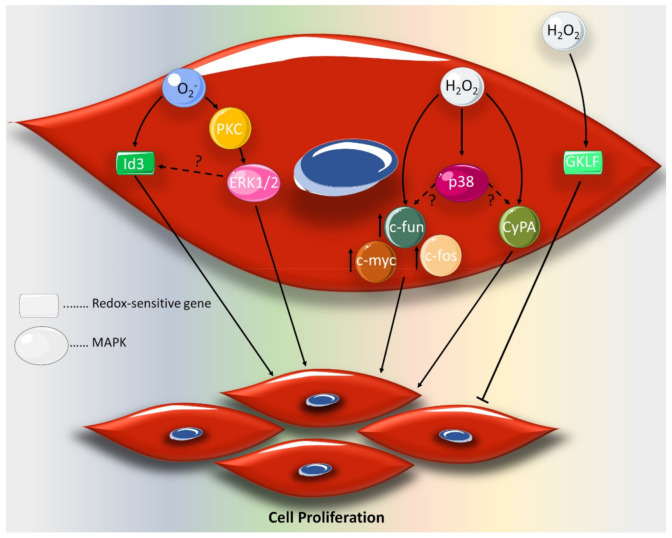
Redox signaling pathways regulating vascular smooth muscle cell (VSMC) proliferation. Superoxide anion, O_2_^−^, induces cell proliferation by activating the mitogen-activated protein kinase (MAPK), ERK1/2, or upregulating the transcription factor Id3. Hydrogen peroxide, H_2_O_2_, promotes VSMC proliferation by activating the p38 MAPK, the CypA chaperone protein and the proto-oncogenes *c-myc*, *c-fos* and *c-jun*. The inhibitory actions of H_2_O_2_ are elicited via the redox sensitive transcription factor gut-enriched Kruppel-like factor, GKLF. Figure key: arrow: activation, block arrow: inhibition, up-arrow: upregulation, question mark: potential crosstalk.

The proliferative effect of ROS may be achieved by activating distinct signaling pathways (Figure 1). For example, O_2_^−^, but not H_2_O_2_, stimulates VSMC proliferation via the rapid PKC-dependent activation of ERK1/2 [49]. Alternatively, O_2_^−^-induced cell proliferation has been reported to be mediated by the dominant negative helix–loop–helix protein, Id3 [56]. On the other hand, cyclophilin A (CyPA), a chaperone protein secreted in response to oxidative stress, dictates the proliferative effect of H_2_O_2_ on VSMCs [57,58]. This H_2_O_2_-induced proliferation is associated with the upregulation of proto-oncogenes *c-myc*, *c-fos* and *c-jun* [59,60]. The aforementioned ROS-induced proliferative effect has been contradicted by some reports. The HO^•^ production by H_2_O_2_ treatment provokes growth arrest by gut-enriched Kruppel-like factor (GKLF) (Figure 1) [56]. The apparent discrepancies may be attributed to the differentially regulated redox sensitive genes, Id3 and GKLF, which provide new insights towards understanding the regulatory effect of ROS on VSMC proliferation and potential differentiation [56]. In addition, the source of ROS, whether endogenous or exogenous, may also contribute to the differential effects of ROS. In agreement with this, it has been shown that treatment with H_2_O_2_ triggers growth arrest [56], while the inhibition of basal H_2_O_2_ attenuates VSMC proliferation [61]. Furthermore, ROS concentration may represent an important factor in determining the role of ROS in VSMC proliferation. Relevantly, H_2_O_2_ induces VSMC proliferation at a concentration of 200 μmol/L [58], but it arrests cell growth at 100 μmol/L [56]. In line with these observations, a 100 μM concentration of H_2_O_2_ has been shown to arrest cell cycle, while the endogenous H_2_O_2_ levels have proven to be crucial for cell proliferation [9]. 

Importantly, the evidence shows that cardiovascular pathologies involving vascular remodeling are accompanied by the upregulation of NOX subunits [62,63,64] and increased ROS release [65,66]. The association of oxidative stress with vascular remodeling reflects a relation between ROS and remodeling events, including migration [43]. Indeed, ROS modulates several events critical for VSMC migration, a characteristic feature of dedifferentiated VSMC [16]. These events include lamellipodia formation, focal adhesion kinase activation and actin polymerization. In response to a certain chemoattractant, ROS mediates Rac-induced actin polymerization, leading to lamellipodia formation [67]. Subsequently, ROS mediates the activation/deactivation of several focal adhesion proteins, which form sites of cell attachment to ECM [43]. Finally, ROS alter actin polymerization by oxidizing the thiols of cytoskeletal reorganization proteins Src [68] and actin [69]. Notably, the rate and the extent of actin polymerization are increased under oxidative conditions [70]. 

In the vasculature, ROS are implicated in the migratory effects of growth factors and hypertrophic hormones [7]. These include phenylephrine, thrombin, vascular epidermal growth factor (VEGF), basic fibroblast growth factor (bFGF), PDGF, insulin-like growth factor-I–induced (IGF-I), and Ag-II [71,72,73]. Moreover, ROS mediate bradykinin-induced VSMC migration and collagen production [53]. Given that the signaling pathways activated by ROS greatly overlap with those driven by the aforementioned ligands, it has been speculated that ROS act as second messengers for growth factors and hypertrophic hormones [40]. Consistently, it has been shown that phenylephrine- and VEGF-induced VSMC migration is mitigated by the antioxidants *N*-acetylcysteine (NAC) and pyrrolidine dithiocarbamate [71,72]. In addition, the NOX inhibitor apocynin attenuates VSMC migration, suggesting that NOXs serve as important mediators in the VSMC migratory signaling pathway [74]. Contextually, NOX4 mediates Ag-II- and IGF-I- induced VSMC migration [75,76], while NOX1 facilitates migration stimulated by bFGF and PDGF [73,77]. 

**Figure 2:**
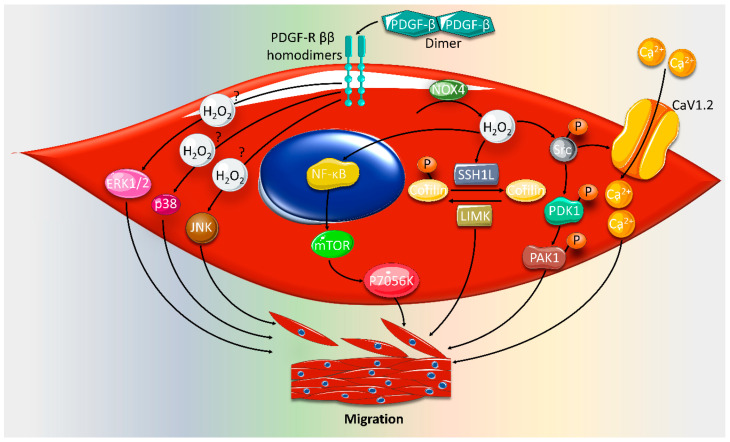
Platelet-derived growth factor-β (PDGF-β)-activated pathways mediating vascular smooth muscle cell (VSMC) migration. After platelet-derived growth factor receptor-β (PDGF-R ββ ) activation, NADPH oxidase-1 (Nox1)-released peroxide (H_2_O_2_) activates Slingshot1L (SSLH1) and LIM kinase (LIMK), cofilin phosphatase and kinase, respectively. The net result is cofilin_._dephosphorylation leading to actin reorganization and ultimately migration. Furthermore, PDGF-induced H_2_O_2_ activates the Src/ (phosphoinositide-dependent kinase-1) PDK1/ (p21-activated protein kinase) PAK1 signaling pathway mediating VSMC migration. Additionally, Src activation increases L-type voltage-dependent calcium channel (CaV1.2) activity leading to increased intracellular calcium (Ca^2+^) concentration, and consequently VSMC migration. In addition, ERK1/2, JNK and p38 mediate PDGF-induced migration. Whether the activation of these MAPKs is ROS-dependent is yet to be determined. Figure key: arrow: activation, question mark: potential crosstalk.

The mechanism of PDGF-induced VSMC migration has been extensively studied (Figure 2). It is mainly mediated by H_2_O_2_ [50,78], and occurs through the PDGF-β receptor [79], as α and β receptors are barely expressed in VSMCs [80,81]. One study showed that ROS mediates PDGF-induced VSMC migration by activating the ROS/NF-κB/mTOR/P70S6K signaling cascade, which also induces VSMC proliferation [82]. In addition, ROS mediates PDGF-induced Slingshot1L (SSH1L) phosphatase and LIM kinase (LIMK) activation [77,83]. In turn, SSH1L and LIMK catalyze cofilin phosphorylation/dephosphorylation, leading to VSMC migration [83]. Interestingly, cofilin is involved in lamellipodium protrusion and actin filaments reorganization, crucial events in VSMC migration [84]. Furthermore, PDGF-induced ROS activate the Src/phosphoinositide-dependent kinase-1 (PDK1)/Rac-effector p21-activated protein kinase (PAK1) signaling pathway [78]. PAK1 activation potentially leads to cytoskeletal rearrangements, and thus facilitates VSMC migration. Src seems to represent a regulatory point from which another pathway diverges. PDGF-activated Src phosphorylates the CaV1.2 channel, leading to an increased intracellular Ca^2+^ concentration. The intracellular Ca^2+^ increase causes actinomyosin rearrangement, culminating in VSMC migration [85]. Finally, the PDGF-mediated migratory signaling cascade is known to comprise the mitogen-activated protein kinases (MAPKs) ERK1/2, JNK and p38 [86]. Whether these pathways are ROS-dependent is still to be determined.

It is worth mentioning that VSMC migration and proliferation is facilitated by matrix degradation and reorganization impelled by matrix metalloproteinases (MMPs) [87,88,89]. Interestingly, MMP expression and activation are regulated by ROS. Indeed, stress-induced ROS upregulate the transcription of MMP-2 [90]. In addition, ROS promote the expression of MMP-2, MMP-9, MMP-14, collagen, fibronectin, integrin α5 and β1 [91]. The secretion of these proteins leads to ECM disorganization, characteristic of synthetic VSMCs [91]. Additionally, ROS activate the pro- MMP-2 and pro-MMP-9 secreted by VSMCs [92]. Remarkably, H_2_O_2_ activates MMP-2 at a concentration of 4 mM, while higher doses (10–50 mM) lead to MMP-2 inactivation [92]. The biphasic response of MMP to oxidative stress may further contribute to our understanding of the differential effects of ROS on VSMC migration and proliferation. 

## 3. Effect of ROS on VSMC Cell Cycle and Cell Fate

Increasing evidence highlights the role of ROS in the VSMC cell cycle and cell fate, whether senescence or apoptosis [93,94]. These events play key roles in the development of atherosclerosis and restenosis [95,96]. Of note, increased VSMC apoptosis and senescence promote plaque rupturing in the atherosclerotic vessels [96,97]. 

**Figure 3:**
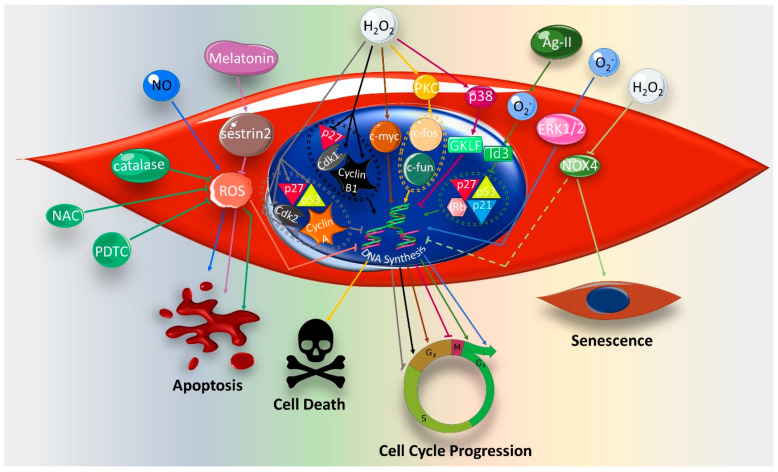
Reactive oxygen species (ROS) regulation of cellular signaling pathways involved in vascular smooth muscle cell (VSMC) fate and cell cycle progression. Superoxides anion, O_2_^−^, -induces DNA synthesis, and consequently cell cycle progression, by activating ERK1/2. Alternatively, O_2_^−^ mediates angiotensin II (Ang-II)-induced cell cycle progression via upregulating Id3 transcription factor, which in turn downregulates cell cycle proteins, including p27, p53 and p21, leading to cell cycle progression. Nitric oxide (NO)-induced ROS induce VSMC apoptosis associated with DNA synthesis inhibition. Hydrogen peroxide (H_2_O_2_) downregulates NADPH oxidase (NOX4), leading to cell senescence and DNA synthesis inhibition. H_2_O_2_ also attenuates cell cycle progression by upregulating the transcription factor gut-enriched Kruppel-like factor (GKLF) via p38. H_2_O_2_ regulates cell cycle proteins and proto-oncogenes to induce cell cycle progression or cell death. Melatonin upregulates sestrin2 leading to ROS inhibition, which decreases VSMC apoptosis. Basal ROS inhibition by catalase overexpression or antioxidants, (Pyrrolidinedithiocarbamate) PDTC or (N-acetylcysteine) NAC, initiates VSMC apoptosis.

Several studies have shown that ROS exhibit an apoptotic effect in VSMC (Figure 3) [98,99]. In this regard, ROS are pivotal mediators of NO-induced apoptosis in VSMCs [100]. Conversely, ROS inhibition by melatonin decreases VSMC apoptosis by upregulation of sestrin2, a ROS scavenger [101]. However, contradictory results regarding the role of ROS in apoptosis have been reported by many studies [59,102]. For instance, it has been shown that basal ROS inhibition by catalase overexpression triggers the apoptosis of rat aortic smooth muscle cells [61,103], indicating that endogenous ROS is important for cell survival and proliferation. Similar results have been obtained upon decreasing ROS levels using the antioxidants pyrrolidinedithiocarbamate (PDTC) and NAC [61,103]. Interestingly, these antioxidants have been used to prevent apoptosis in other cell types, such as lymphocytes, neurons and vascular endothelial cells [104,105,106], suggestive of the cell-type-specific effects of ROS [103]. Taken together, these results selectively highlight the potential benefits of ROS suppression in an atherosclerotic setting.

ROS have been depicted as critical regulators of DNA synthesis in VSMC (Figure 3). In this context, O_2_^−^ induces DNA synthesis in VSMC, leading to cell proliferation [49]. Likewise, H_2_O_2_ provokes DNA synthesis in rat aortic VSMCs by upregulating *c-fos* and *c-jun* via the PKC and PLA2 release of arachidonic acid [107,108]. However, in contrast to O_2_^−^, H_2_O_2_-induced DNA synthesis results in VSMC death [109]. This has been explained by the fact that H_2_O_2_ downregulates α, δ and ϵ isoforms, but upregulates the ζ isoform of PKC, which stimulates DNA synthesis [109]. The stimulatory effect of H_2_O_2_ on cell cycle progression and VSMC proliferation has been reported elsewhere, with the implication of several intracellular proteins as mediators [110]. These include an upregulated cyclin B1 and cyclin-dependent kinase (Cdk1), and a downregulated cyclin-dependent kinase inhibitor, Kip1/p27 [110]. Concomitantly, an increased expression of *c-myc*, an oncogene that promotes cell growth, has been shown to associate with H_2_O_2_-induced VSMC proliferation [110]. On the contrary, H_2_O_2_ has been reported to trigger cell cycle arrest by inhibiting Cdk2 and cyclin A, and upregulating the cell cycle inhibitors p21 and p53 [99]. Thus, H_2_O_2_, by activating various signaling pathways, may positively or negatively regulate the cell cycle, leading to different cellular events. 

Accumulating evidence demonstrates a role for ROS in VSMC senescence. In fact, the treatment of human primary VSMCs with H_2_O_2_ induces their premature senescence (Figure 3), which is accompanied by a decreased expression of ROS-producing NADPH oxidase, NOX4 [111]. The attenuated activity or expression of NOX4 results in not only cellular senescence but also the secretion of pro-inflammatory cytokines [111]. Thus, further research must be conducted to elucidate the interplay between ROS and NOX4 in inducing senescence in VSMC. In addition, the safety of using NOX4 inhibitors in treating redox-related vascular diseases should be assessed. 

**Figure 4:**
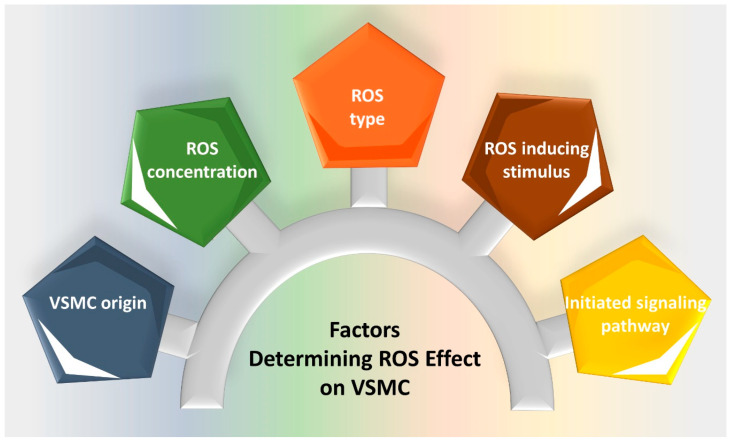
Various factors determining the effect of ROS on VSMC fate. ROS-inducing stimulus, ROS concentrations, as well as ROS type, play a major role in VSMC response. ROS-initiated signaling pathways involving redox-sensitive genes underwrite VSMC cell fate regulation. The vascular beds from which VSMCs are isolated contribute to their differential responses to ROS.

**Figure 5:**
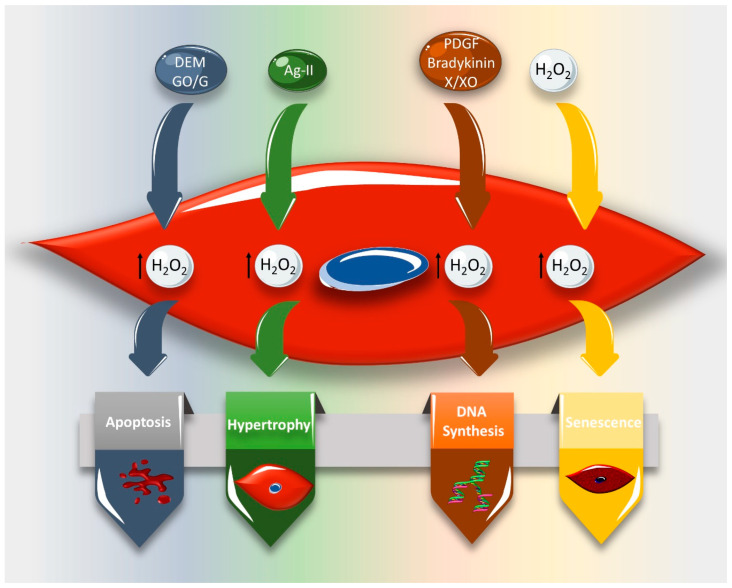
Diverse vascular smooth muscle cell (VSMC) responses to different hydrogen peroxide (H_2_O_2_)-generating stimuli. Glucose oxidase/glucose (GO/G)- or diethylmaleate (DEM)-induced H_2_O_2_ leads to VSMC apoptosis, while angiotensin II (Ang II)-induced H_2_O_2_ causes VSMC hypertrophy. H_2_O_2_ released in response to xanthine/xanthine oxidase, platelet-derived growth factor (PDGF), or bradykinin promotes DNA synthesis and, consequently, VSMC proliferation. Treating human VSMCs with H_2_O_2_ induces cell senescence. Figure key: up-arrow: increment.

The apparent discrepancy in ROS-induced cell fate may be due to several factors, including ROS species, processing and concentration (Figure 4) [40]. While moderate H_2_O_2_ concentrations (100 μmol/L) promote cell cycle arrest, high concentrations (500 μmol/L to 1 mmol/L) lead to apoptosis [99]. Furthermore, the level of redox state is another contributing factor, determining the effect of ROS on VSMC [112]. While the basic ROS level appears to promote VSMC proliferation [7,103], a high oxidative stress setting paradoxically induces cell death [112]. In addition, the VSMC source seems to be a key determinant of the response to ROS. Treatment with H_2_O_2_ (100 μmol/L) induces the senescence of human primary VSMCs [111], while the same concentration provokes the cell proliferation of rat aortic VSMCs [110]. Moreover, the ROS-activated pathway probably depends on the ROS-generating stimulus, as well as their source and localization (Figure 5) [39,113]. Whereas H_2_O_2_ mediates glucose oxidase/glucose (GO/G) or diethylmaleate (DEM)-induced VSMC apoptosis [94], the Ang II-induced H_2_O_2_ leads to VSMC hypertrophy, a hallmark of many vascular diseases [112]. Furthermore, xanthine/xanthine oxidase-produced H_2_O_2_ induces DNA synthesis via the PKC-upregulated expression of proto-oncogenes *c-myc* and *c-fos* [59]. A similar proliferative effect is promoted by PDGF- and braydakinin-induced H_2_O_2_ [50,53]. The presence of redox-sensitive genes in VSMCs adds another level of complexity to the effect of ROS on these cells. An interesting example is the differential activation of genes encoding for the redox-sensitive transcription factors Id3 and GKLF [56,114]. In this respect, Ang II-induced O_2_^−^ increases the expression of Id3, which in turn inhibits the transcription factor E2A, resulting in cell cycle progression [114]. This was accompanied by the downregulation of cell cycle proteins p21^WAF1/Cip1^, p27^Kip1^, p53 and Rb [114]. On the other hand, H_2_O_2_ activates p38, which in turn upregulates GKLF, leading to cell cycle arrest [56] (Figure 3). 

## 4. Effect of ROS on VSMC Differentiation Markers

It is well-established that ROS have a direct effect on VSMC phenotypic plasticity. Several lines of evidence show that ROS can induce contractile, synthetic and osteogenic phenotypes. The variation in the ROS-induced phenotypes appears to be dependent on the nature of the VSMC microenvironment—whether quiescent, differentiating, atherosclerotic or diabetic.

**Figure 6:**
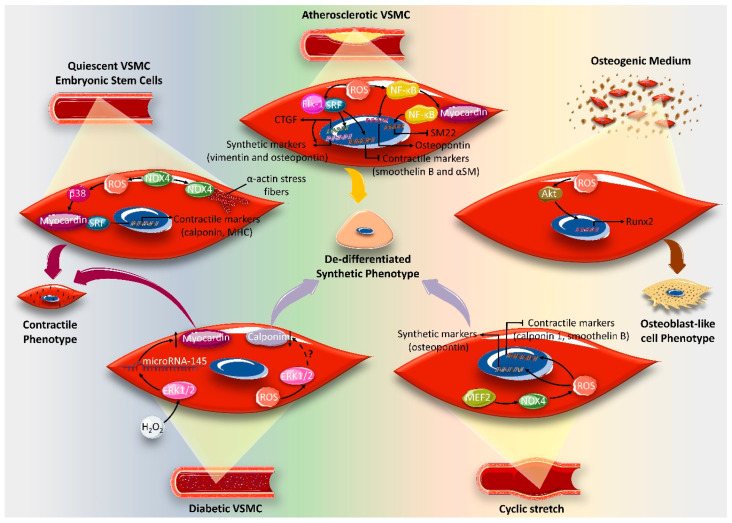
The impact of the vascular smooth muscle cell (VSMC) microenvironment on determining the effect of reactive oxygen species (ROS) on phenotypic switch. Oxidative stress seems to be crucial for maintaining the contractile phenotype of quiescent VSMCs and for the differentiation of embryonic contractile VSMCs. NAPDH oxidase (NOX4)-produced hydrogen peroxide (H_2_O_2_) activates the transcription complex serum response factor (SRF)/myocardin via p38. This complex translocates to the nucleus and upregulates the transcription of contractile markers, such as calponin and myosin heavy chain. NOX4 also seems to play a structural role in maintaining a contractile phenotype by binding to α-actin stress fibers, characteristic of this phenotype. In the context of atherosclerosis, ROS induces VSMC dedifferentiation by activating the NF-κB and/or Elk-1/SRF signaling pathways. NF-κB upregulates the transcription of the synthetic marker osteopontin, and associates with myocardin to repress the myocardin-dependent contractile gene expression of smooth muscle 22 (SM22). Alternatively, ROS promote Elk-1/SRF complex formation, which activates the transcription of synthetic markers, vimentin and osteopontin, via connective tissue growth factor (CTGF). The Elk-1/SRF complex, alternatively, downregulates the contractile markers smoothelin B and α-smooth muscle (αSM). In an osteogenic medium, ROS induce VSMCs, which induces transition to the osteoblast-like cell phenotype, characteristic of vessel calcification in advanced atherosclerosis. The ROS-induced osteoblast-like cell phenotype is mediated via AKT-activated Runx2, a key transcription factor for osteogenic differentiation. In diabetic VSMC, ROS induce a synthetic phenotype by decreasing calponin, probably via ERK1/2. Conversely, ROS provoke the contractile phenotypic switch of diabetic VSMC by upregulating microRNA-145, which in turn increases the activity of myocardin in an ERK1/2-dependent manner. Cyclic stretch evokes a VSMC synthetic phenotypic switch through NOX1-derived ROS release via myocyte enhancer factor 2B (MEF2B), resulting in the upregulation of osteopontin and the downregulation of contractile markers calponin1 and smoothelin B. Figure key: up-arrow: upregulation, down-arrow: downregulation, arrow: activation, block arrow: inhibition, question mark: potential crosstalk.

In quiescent VSMCs, oxidant activity is required for the increased expression of differentiation markers, including calponin, smooth muscle (SM1 and SM2) myosins and α-actin (Figure 6) [41]. This ROS-induced upregulation is mediated via p38 [41]. Given that p38 increases the transcriptional activity of the SMC-specific transcription factor serum response factor (SRF) [115,116], the potential involvement of SRF in the ROS/p38-mediated increase in VSMC differentiation seems likely [41]. It is worth mentioning that ROS in quiescent VSMCs are derived from NOX4 [117]. In addition to its functional role in inducing a contractile phenotype, Nox4 is thought to play a structural role by maintaining VSMC differentiation [118]. This role stems from the observation that during the early de-differentiation process, Nox4 translocates from the α-actin stress fibers in contractile VSMCs to focal adhesions in de-differentiated cells [118,119]. Interestingly, an attenuated Nox4 activity decreases the level of endogenous H_2_O_2_, and induces a synthetic VSMC phenotype and increased ECM deposition [119]. Thus it appears that a basal level of ROS is needed to maintain a contractile phenotype.

Another niche where ROS induces contractile gene expression is during the differentiation of embryonic stem cells to VSMCs (Figure 6) [42]. Indeed, NOX4-produced H_2_O_2_ induces the activation of SRF, which translocates to the nucleus and recruits the muscle-specific co-activator myocardin [42]. The resulting SRF/myocardin complex binds to the promoter-enhancing region of the VSMC contractile marker genes, such as calponin and myosin heavy chain, and induces their expression [120]. 

In the context of atherosclerosis, SRF plays the opposite role by attenuating the expression of contractile markers genes [121], and promoting VSMC dedifferentiation (Figure 6). In fact, SRF may bind to one of two competing partners, myocardin or Elk-1. Whereas the SRF–myocardin complex induces the transcription of smooth muscle differentiation genes, the SRF–Elk-1 complex represses the transcription of these genes [91]. Interestingly, ELK-1 is greatly upregulated in thoracic aortic aneurysm (TAA) tissues and is undetectable in normal aortic tissues [91]. As such, in TAA patients, ROS rather induce the VSMC synthetic phenotype via the Elk-1/SRF signaling pathway, by upregulating the connective tissue growth factor (CTGF) [91]. The VSMC synthetic induction is accompanied by an increase in the dedifferentiation markers, osteopontin and vimentin, as well as a decrease in the contractile markers, smoothelin B and smooth muscle α [91]. Alternatively, in response to inflammatory cues, ROS may induce VSMC dedifferentiation by activating the key transcriptional factor, NF-κB [53] (Figure 6). NF-κB upregulates the transcription of osteopontin [122], a glycoptrotein involved in the phenotypic transition of VSMCs towards the synthetic phenotype [123]. Additionally, NF-κB suppresses the myocardin-dependent expression of the contractile marker, SM22 [124]. 

Advanced stages of atherosclerosis are characterized by vascular calcification [125]. Notably, ROS, namely H_2_O_2_, have the potential to promote calcification by inducing the VSMC switch to an osteoblast-like cell phenotype in a defined osteogenic medium [126]. This H_2_O_2_-induced phenotypic switch is mediated via AKT-activated Runx2, a key transcription factor for osteogenic differentiation [126].

Under diabetic conditions, excessive ROS production affects the VSMC phenotype [95,127,128]. Mesenteric VSMCs from type 2 diabetic Goto-Kakizaki rats show a decreased expression of calponin (Figure 6) [128]. The downregulation of this contractile marker is associated with increased ROS levels and enhanced ERK1/2 activation [128]. However, in aortic VSMCs extracted from the same diabetic model, treatment with H_2_O_2_ upregulates microRNA-145, which increases the activity of myocardin, thus inducing contractile gene transcription [127]. These effects are mediated via ERK1/2 [127]. The inconsistency in diabetic VSMC redox response could be explained by the distinct VSMC origin—whether extracted from large vessels or microvessels. 

Moreover, VSMC phenotypic switch has been reported to be influenced by microenvironment mechanical cues, specifically cyclic stretch [25]. In fact, cyclic stretch upregulates myocyte enhancer factor 2B (MEF2), a transcription factor known for its role in cell proliferation and differentiation [129]. MEF2B then potentiates NOX1-mediated ROS release, leading to VSMC phenotypic switch, as evidenced by the upregulation of osteopontin and the downregulation of contractile markers, calponin1 and smoothelin B [25].

In addition to VSMC microenvironment, the differential phenotypic response to ROS may be explained by other inhibitory pathways that may override the stimulatory effect of ROS [41]. For instance, although PDGF increases ROS in VSMCs [50], it attenuates the expression of differentiation markers [130]. As a consequence, the PDGF-generated high oxidant activity in proliferating VSMCs fails to stimulate differentiation [41]. Congruently, increased H_2_O_2_ production decreases and increases the expression of contractile and synthetic markers, respectively [131]. 

## 5. ROS and VSMC Epigenetics 

Evidence shows that epigenetic modifications regulate the phenotype of VSMCs [132,133]. These alterations, including DNA modifications, histone modifications and ATP-dependent chromatin remodeling, affect the gene expression pattern contributing to the VSMC phenotypic switch [134]. For instance, the histone modifying enzymes, histone acetyltransferases (HATs) and histone deacetylases (HDACs), potentially play a role in atherosclerosis and restenosis after coronary intervention by promoting neointima formation, smooth muscle cell proliferation and inflammation [135]. In addition, an increased susceptibility to atherosclerotic changes in the VSMCs of ApoE+/− mice is associated with altered histone methylation and lysine methyltransferase expression [136]. Notably, epigenetic pathways in the nuclear and mitochondrial genome involve ROS as signaling messengers [134]. Here, we present some reports that support the role of ROS in coordinating the effects of epigenetic modifications in VSMC phenotypic switch. 

ROS is known to affect DNA methylation by targeting the expression and/or activity of the DNA-modifying enzyme ten-eleven translocation-2 (TET2) [134,137,138]. In human atherosclerosis and in a mouse model of vascular injury, conditions known for their increased oxidative stress, the activity of TET2 is reduced in VSMCs, contributing to their switch to the synthetic phenotype [133]. Thus, it is tempting to speculate that TET2 activity is reduced by the increased ROS level in atherosclerotic VSMCs. Furthermore, proliferating VSMCs exhibit decreased whole genome methylation content in atherosclerotic aortas as compared with normal arteries [139]. VSMCs in atherosclerotic plaque show also a reduced methylation in the extracellular superoxide dismutase (EC-SOD) gene [139]. Although these observations do not provide a causal relationship between hypomethylation and atherosclerosis, they unquestionably reveal that EC-SOD hypomethylation is associated with atherosclerosis [139] and consequently VSMC phenotypic switch [139,140]. Given that ROS are increased in atherosclerosis and regulate SOD [141], it is plausible to hypothesize that ROS signaling is involved in the hypomethylation profile of SOD, and potentially in the whole genome. 

Histone modifications contribute to VSMC’s phenotypic switch during atherosclerosis and intimal injury [142]. In response to these conditions, SRF causes post-translational histone modifications, suppressing smooth muscle cell differentiation [121]. Knowing that atherosclerosis and intimal injury are characterized by high levels of ROS and that ROS regulate SRF, it can be assumed that ROS are potentially implicated in the SRF-induced suppression of VSMC differentiation markers. 

## 6. Conclusions

It is now evident that a basal ROS level is crucial for vascular homeostasis, particularly in the maintenance of VSMC survival, contractility and intracellular signaling [143]. However, the dysregulation in ROS generating- and/or ROS-scavenging enzymes leads to oxidative stress, which induces VSMC proliferation and dedifferentiation [9]. The VSMC phenotypic switch is implicated in the pathogenesis of vascular diseases [9]. Thus, it is reasonable to assume that antioxidants play a vasculoprotective role. In support of this assumption, clinical trials have shown that administering antioxidants such as vitamin C, vitamin E and resveratrol improves vascular function [144,145,146,147]. On the other hand, other clinical trials have not succeeded in finding a relation between antioxidants consumption and cardiovascular morbidity [148,149,150,151]. The failure of antioxidants to grant cardiovascular protection could be ascribed to several factors, pertinent to the antioxidant agent used, such as its pharmacokinetics, dosage, efficacy and selective scavenging activity [9,152,153,154], and/or to the patients, such as their age, disease stage and presence of oxidative stress [9]. 

To date, there is no well-defined therapeutic strategy for the clinical prescription of antioxidants [143], as the results of clinical trials in this aspect are not encouraging. In addition, caution must be taken when using antioxidants, as they are able to play a pro-oxidant role by reducing iron ions [155]. Iron reduction by antioxidants leads to ROS production, resulting in oxidative damage to proteins, lipids and DNA [156,157]. Another cautionary note is that antioxidants may halt vital ROS-dependent signaling pathways [143]. For instance, cold-induced vasoconstriction is mediated by the α_2C_-adrenergic receptor, a receptor functionally rescued by ROS [158]. Thus, more investigations and research must be conducted to specify the nature of the ROS implicated in a given CVD, on the one hand, and to augment the efficiency of antioxidants on the other hand.

## Abbreviations

Ag-II	Angiotensin II
bFGF	Basic fibroblast growth factor
Cdk	Cyclin-dependent kinase
CTGF	Connective tissue growth factor
CVD	Cardiovascular disease
CyPA	Cyclophilin A
ECM	Extracellular matrix
EC-SOD	Extracellular superoxide dismutase
FGF	Fibroblast growth factor
GKLF	Gut-enriched Kruppel-like factor
H_2_O_2_	Hydrogen peroxide
HAT	Acetyltransferase
HDAC	Histone deacetylase
HO^•^	Hydroxyl radical
IGF-I	Insulin-like growth factor-I
LIMK	LIM kinase
MEF2B	Myocyte enhancer factor 2B
MCP-1	Monocyte chemokine protein 1
MMP	Matrix metalloproteinases
NAC	N-acetylcysteine
NO	Nitric oxide
NOX	NADPH Oxidase
O_2_^−^	Superoxide anion
PAK1	Rac-effector p21-activated protein kinase
PDGF	Platelet-derived growth factor
PDK-1	Phosphoinositide-dependent kinase-1
PTDC	Pyrrolidinedithiocarbamate
TGF-β	Transforming growth factor
ROS	Reactive oxygen species
SM	Smooth muscle
SOD	Superoxide dismutase
SRF	Serum response factor
SSHL1	Slingshot1L
TAA	Thoracic aortic aneurysm
TET2	Ten-eleven translocation-2
VEGF	Vascular epidermal growth factor
VSMCs	Vascular smooth muscle cells
